# Continuous Non-invasive finger cuff CareTaker® comparable to invasive intra-arterial pressure in patients undergoing major intra-abdominal surgery

**DOI:** 10.1186/s12871-017-0337-z

**Published:** 2017-03-21

**Authors:** Irwin Gratz, Edward Deal, Francis Spitz, Martin Baruch, I. Elaine Allen, Julia E. Seaman, Erin Pukenas, Smith Jean

**Affiliations:** 1 Department of Anesthesiology, Cooper Medical School at Rowan University Cooper University Hospital, Camden, New Jersey USA; 2 Empirical Technologies Corporation, Charlottesville, Virginia USA; 3 Department of Biostatistics and Epidemiology, University of California, San Francisco, CA USA; 4 Department of Pharmaceutical Chemistry, University of California, San Francisco, California USA

**Keywords:** Non-Invasive, CareTaker, Central blood pressure, Finger cuff, Intra-Arterial pressure

## Abstract

**Background:**

Despite increased interest in non-invasive arterial pressure monitoring, the majority of commercially available technologies have failed to satisfy the limits established for the validation of automatic arterial pressure monitoring by the Association for the Advancement of Medical Instrumentation (AAMI). According to the ANSI/AAMI/ISO 81060–2:2013 standards, the group-average accuracy and precision are defined as acceptable if bias is not greater than 5 mmHg and standard deviation is not greater than 8 mmHg. In this study, these standards are used to evaluate the CareTaker® (CT) device, a device measuring continuous non-invasive blood pressure via a pulse contour algorithm called Pulse Decomposition Analysis.

**Methods:**

A convenience sample of 24 patients scheduled for major abdominal surgery were consented to participate in this IRB approved pilot study. Each patient was monitored with a radial arterial catheter and CT using a finger cuff applied to the contralateral thumb. Hemodynamic variables were measured and analyzed from both devices for the first thirty minutes of the surgical procedure including the induction of anesthesia. The mean arterial pressure (MAP), systolic and diastolic blood pressures continuously collected from the arterial catheter and CT were compared. Pearson correlation coefficients were calculated between arterial catheter and CT blood pressure measurements, a Bland-Altman analysis, and polar and 4Q plots were created.

**Results:**

The correlation of systolic, diastolic, and mean arterial pressures were 0.92, 0.86, 0.91, respectively (*p <* 0.0001 for all the comparisons). The Bland-Altman comparison yielded a bias (as measured by overall mean difference) of −0.57, −2.52, 1.01 mmHg for systolic, diastolic, and mean arterial pressures, respectively with a standard deviation of 7.34, 6.47, 5.33 mmHg for systolic, diastolic, and mean arterial pressures, respectively (*p <* 0.001 for all comparisons). The polar plot indicates little bias between the two methods (90%/95% CI at 31.5°/52°, respectively, overall bias = 1.5°) with only a small percentage of points outside these lines. The 4Q plot indicates good concordance and no bias between the methods.

**Conclusions:**

In this study, blood pressure measured using the non-invasive CT device was shown to correlate well with the arterial catheter measurements. Larger studies are needed to confirm these results in more varied settings. Most patients exhibited very good agreement between methods. Results were well within the limits established for the validation of automatic arterial pressure monitoring by the AAMI.

## Background

Accurate real-time continuous non-invasive blood pressure monitors (cNIBP) can bridge the gap between invasive arterial pressure monitoring and intermittent non-invasive sphygmomanometry. Latest developments in this field promise accuracy and the potential to lower risk and improve patient outcomes. However, a recent systematic review and meta-analysis of 28 studies using non-invasive technologies by Kim et al. reported that all failed to satisfy the limits that have been established for the validation of automatic arterial pressure monitoring by the Association for the Advancement of Medical Instrumentation (AAMI) [1]. According to this standard, the group-average accuracy and precision are defined as acceptable if bias is not greater than 5 mmHg and standard deviation is not greater than 8 mmHg. Kim et.al. obtained similar results when currently commercially available technologies were examined [1]. In addition, ease of use and patient comfort issues have been impediments to wider acceptance of current noninvasive cNIBP measurement methods. Their results suggest that currently available devices may not have the accuracy and precision for reliable clinical decisions, and there is a need for better devices.

**Fig. 1:**
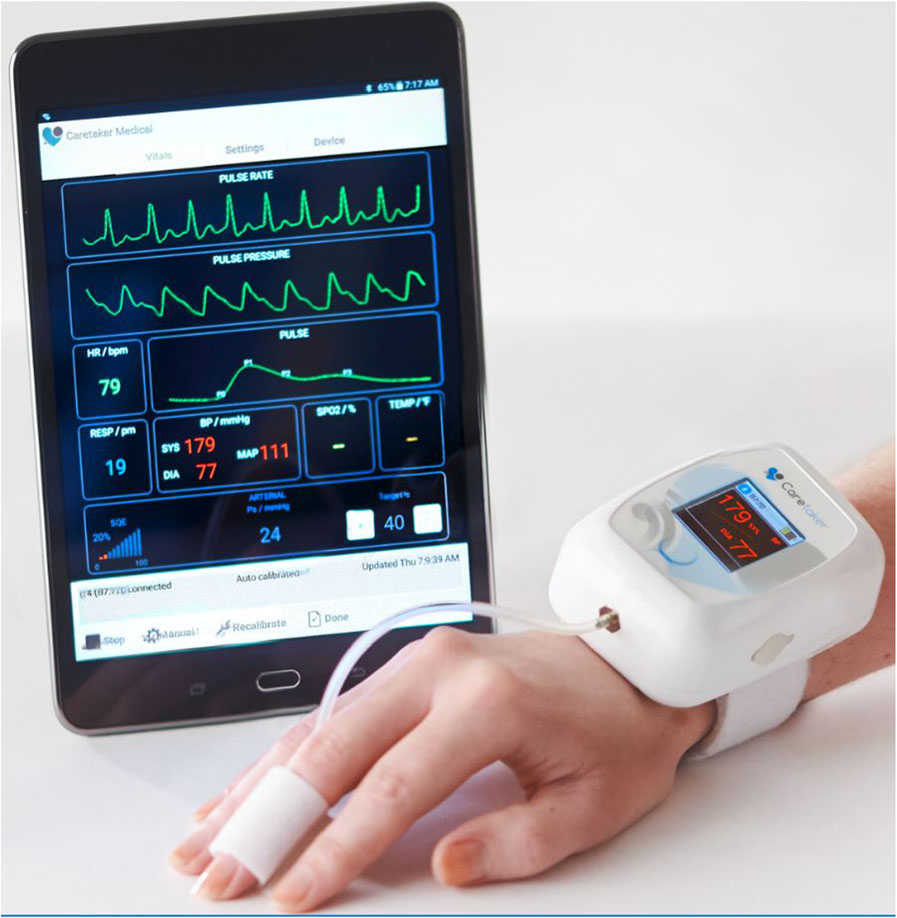
CareTaker Wireless Continuous Blood Pressure and Heart Rate Monitor with Finger Cuff Technology. Copyright 2016. Used with written permission from president and CEO of CareTaker Medical, LLC

We evaluated the CareTaker® (CT) device (Empirical Technologies Corporation, Charlottesville, Virginia) which has been described in detail elsewhere [2]. Briefly, the CT is a physiological sensing system that communicates physiological data wirelessly via Bluetooth (Fig. 1). The device uses a low pressure [35–45 mmHg], pump-inflated, cuff surrounding the proximal phalange of the thumb that pneumatically couples arterial pulsations via a pressure line to a custom-designed piezo-electric pressure sensor. This sensor converts the pressure pulsations, using transimpedance amplification, into a derivative voltage signal that is then digitized at 500 Hz, transmitted to and recorded on a computer.

**Fig. 2:**
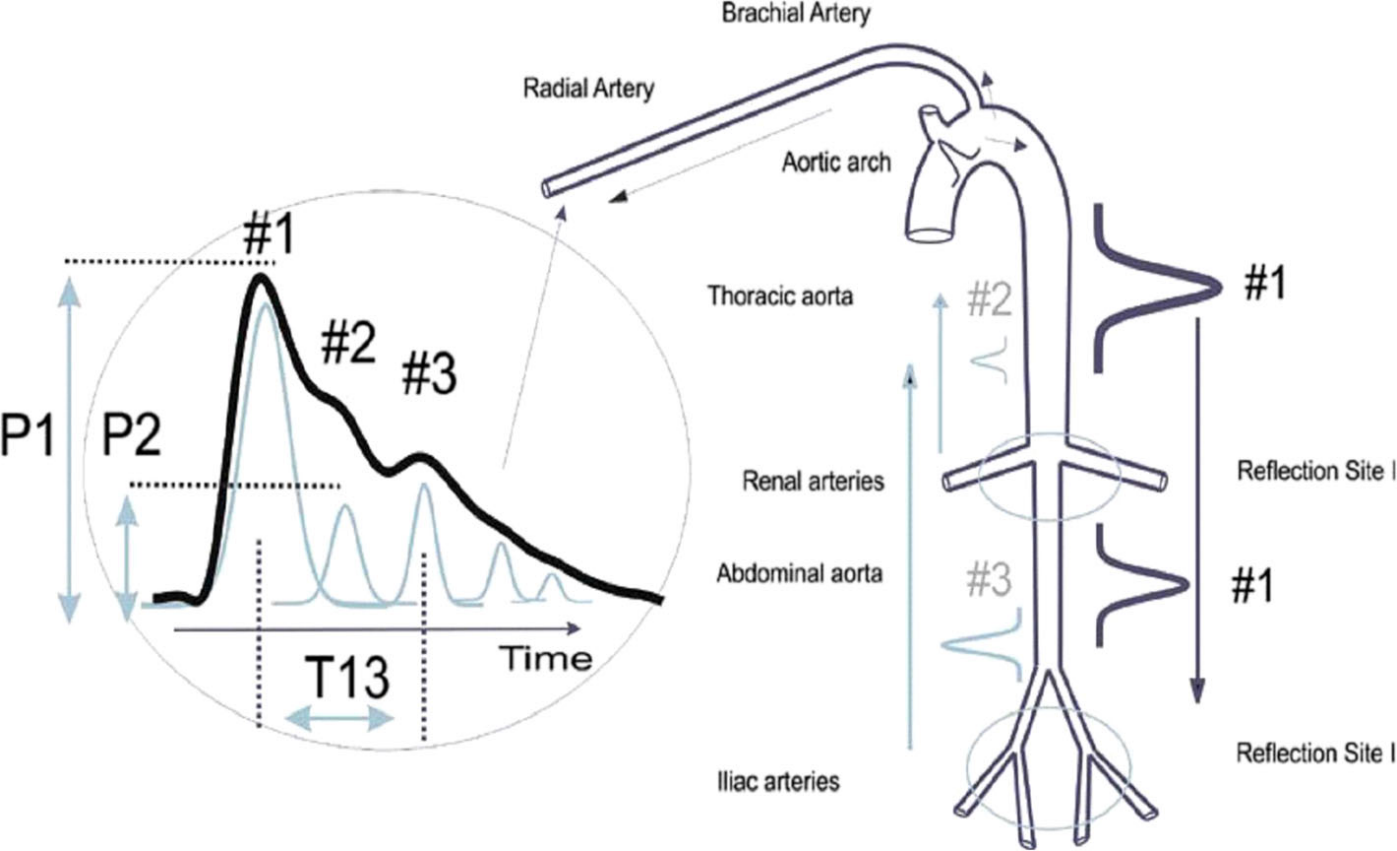
Sketch of the aorta/arm complex arterial system and its effect on the arterial pressure pulse line shape that is observed at the radial/digital artery. Two reflection sites, one at the height of the renal arteries, the other one in the vicinity of the iliac bifurcation, give rise to the reflected pulses (*gray*) that trail the primary left ventricular ejection (*black*). Amplitudinal changes between the left ventricular ejection pulse P1 and the renal reflection pulse P2 as well as timing changes between P1 and the iliac reflection P3 are used to track blood pressure. An arterial stiffness measure is derived from the inversion profile of the pulse envelope

The CT measures continuous noninvasive blood pressure via a pulse contour analysis algorithm called Pulse Decomposition Analysis (PDA) [3]. It is based on the concept that five individual component pressure pulses constitute the peripheral arterial pressure pulse. These component pulses are due to the left ventricular ejection and the reflections and re-reflections of the first component pulse from two central arteries reflection sites [2] [4]. The first reflection site is the juncture between thoracic and abdominal aorta, at the height of the renal arteries, while the second site arises from the interface between abdominal aorta and the common iliac arteries. The renal site reflects the pressure pulse because the juncture of the aortic arteries there features significant changes in arterial diameter and wall elasticity. The two reflected arterial component pressure pulses, the renal reflection pulse (P2) and the iliac reflection pulse (P3), counter-propagate with respect to the original pulse due to the left ventricular contraction (Fig. 2) and arrive in the arterial periphery, specifically at the radial or digital arteries, with distinct time delays [5]. The basic validity of the PDA model was recently corroborated in a detailed and comprehensive arterial tree numerical modeling analysis [6] that examined the effect of the different arterial segments of the central arteries, the iliac arteries and beyond on the pressure/flow pulse patterns in the digital arteries. The results clearly identified the central arterial reflection sites, as opposed to more distal sites, as being the primary contributors to the pulse patterns observed in the digits.

Quantification and validation of physiological parameters is accomplished by extracting pertinent component pulse parameters [7]. Since the device relies on pulse analysis to track blood pressure, the coupling pressure of the finger cuff is maintained constant and well below diastole, avoiding potential blood flow impediments.

The aim of the present study was to specifically compare the non-invasive arterial pressure values obtained with the CT to the reference invasive arterial pressure technique.

## Methods

The Cooper Health System Institutional Review Board approved the study, and all subjects gave informed written consent. Data from twenty-four adult patients requiring hemodynamic monitoring during major open abdominal surgery were analyzed in this study. Patients were not excluded due to other medical conditions.

Measurements were obtained during general anesthesia in these patients starting with induction. The induction of anesthesia was chosen because the blood pressure fluctuations and variability typically found during this period provided an opportunity to compare tracking accuracy under baseline and induced controlled dynamic conditions. The data was evaluated using the ANSI/AAMI/ISO 81060–2:2013-related standards of accuracy and precision [8].

### Anesthesia procedure

After a stable signal was recorded, patients were induced under general anesthesia by using propofol (2-4 mg/kg) and fentanyl 250ug. Tracheal intubation was facilitated by the administration of rocuronium (0.6 mg/kg). Mechanical ventilation was started using a volume controlled ventilator to maintain an adequate saturation and an end-tidal carbon dioxide of 35 mmHg. Inhalational anesthetic (Isoflurane) was added to maintain a BIS monitoring of 40–45. Vasoactive drugs were used to maintain a MAP greater than 60 mmHg based on the catheter value. Hemodynamic variables were measured from both devices for the entire procedure. The MAP, systolic and diastolic blood pressures were continuously collected from the arterial catheter and CT and averaged over 10 s periods for both devices.

### Invasive arterial pressure measurement

Standard arterial blood pressure monitoring was performed prior to the induction of anesthesia using a 20G intra-arterial catheter inserted in the radial artery under local anesthesia using ultra sound guidance. The catheter was connected to a disposable pressure transducer with standard low compliant tubing. The transducer was placed at heart level and zeroed to ambient pressure. The transducer data was digitized, processed and collected using the Datex-Ohmeda S/5 Collect system (Datex-Ohmeda Division, Instrumentarium Corporation, Helsinki, Finland). For analysis, MAP, systolic and diastolic blood pressures were averaged over 10 s intervals.

### Non-invasive CareTaker arterial pulse signal recording

The arterial pressure pulse signal was continuously measured using the CT device. For this study the CT device was calibrated using the arterial line blood pressure, but calibration can also be based on non-invasive oscillometric or oscillometric/auscultatory measurements. A fifteen second window at the start of the 30 min overlap section was used to obtain an arterial stiffness reading averaged across 5 beats, which was then used to calculate the PDA parameters for the blood pressure conversions (Fig. 2). With the exception of the four cases mentioned above, patient-specific PDA parameters, once established, were not changed for the matching procedure, irrespective of arterial stiffness or heart rate changes. On four occasions for the entire data set, the offsets of the linear conversion equations were changed as a result of persistent changes in arterial stiffness or heart rate changes exceeding 30%. The PDA algorithm has recently been validated and described elsewhere [6].

### Data inclusion

Arterial catheter data were visually inspected and sections of obvious catheter failure, characterized by either continuous or spurious nonsensical reading, were excluded. Sections contaminated by excessive motion artifact such that the peak detection algorithm was no longer able to identify heart beats were also excluded. In the case of the CT data, a custom signal/noise factor (SNF) was used to identify poor quality data sections which were excluded. The factor is based on the standard ratio of the variances of the physiological signal band to the noise band and obtained using Fourier spectral analysis over an 8-s window with 1 s overlap [9]. The frequency range of the band associated with the physiological signal was set to 1–10 Hz, based on data by the authors and results by others, [7] while the noise band was set to the 100–250 Hz frequency range, which is subject to ambient noise but contains no signal relevant to the base band phenomena of the arterial pressure pulse or its propagation characteristics. Data sections with an SNF below 80 were excluded from the analysis.

### Comparisons of the two methodologies

All comparisons between CT data and arterial catheter data were post-processed. For each patient, the first 30 min overlap section was used for the comparison. A stable overlap section was defined as having an SNF of at least 140 for the CT data and having stable a-line data, as described above. In a onetime procedure, a 15 s window at the start of the 30 min overlap section was used to obtain PDA pulse parameters averaged across 5 beats which were then used for the blood pressure conversions. Patient-specific PDA parameters, once established, were not changed for the matching procedure, irrespective of hemodynamic changes.

### Statistical analysis

Initially, the data were examined to ensure that each method did not depart significantly from the normal distribution using the Shapiro-Wilk test. Intra- and inter-patient differences were calculated using matched datasets. To compare the two methods, Bland-Altman plots with corresponding correlation coefficients and Pitman test results were constructed for systolic, diastolic and the MAP. The 95% confidence intervals were calculated for each plot.

Because the estimation of the difference between the methods was the outcome of interest, no power analyses for sample size estimates were calculated prior to the study. Initial cohort size of 24 was therefore primarily driven by patient availability and the 81060 standard’s required lower limit of 15 patients when an a-line is used for comparison (http://www.scholarpedia.org/article/Signal-to-noise_ratio). Further comparison of the methods was done with a 4-Quadrant plot and polar plot. For the 4-Quadrant plot, differences in successive measurements for each device were plotted to compare the agreement in magnitude and direction of values [10]. Concordance and angular bias were calculated. A polar plot was computed from the data to examine any bias in the comparison between the A-line and the CT device [11]. The values in the center of the plot show close agreement between the A-line and the CT monitors and are excluded from trend analyses [12]. Confidence intervals (95% and 90%) were calculated and shown as radians between dashed lines from the center of the plot. Between patient variability was examined using general linear models controlling for time of measurement during surgery. Statistical analyses were performed in Stata 13.2 (StataCorp, College Station, TX) and R (https://cran.r-project.org/).

## Results

**Fig. 3:**
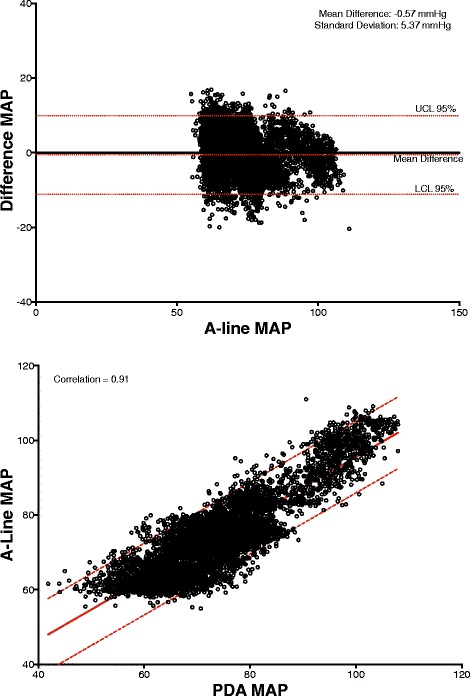
(*Top*) Bland Altman graphs of MAP difference vs. A-line for all timepoints. Correlation (*bottom* graph, linear fit with 95% confidence bounds)

**Fig. 4:**
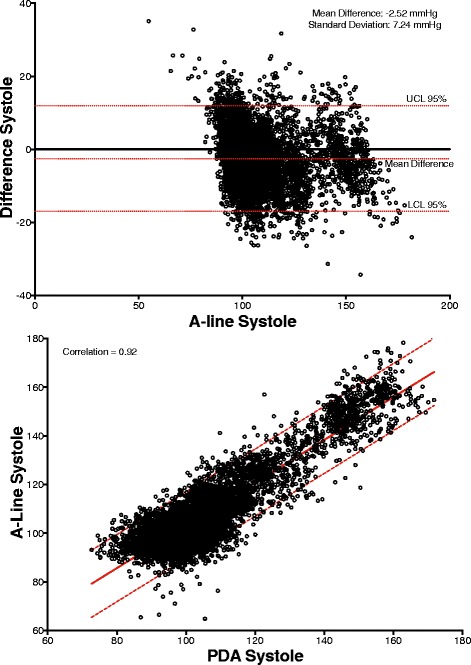
(*Top*) Bland Altman graphs of Systole difference vs. A-line for all timepoints. Correlation (*bottom* graph, linear fit with 95% confidence bounds)

**Fig. 5:**
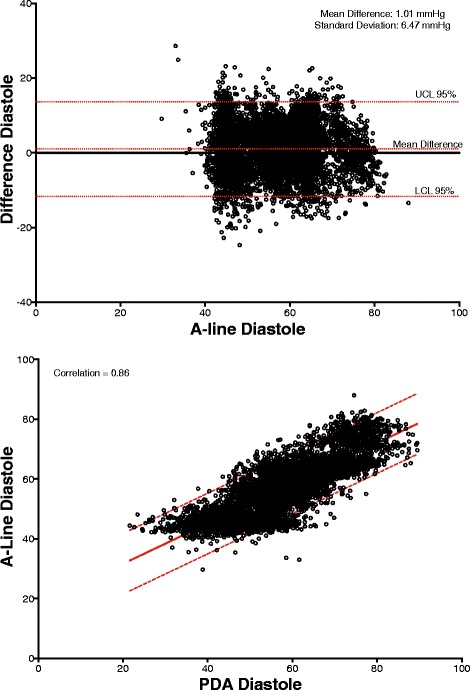
(*Top*) Bland Altman graphs of Diastole difference vs. A-line for all timepoints. Correlation (*bottom* graph, linear fit with 95% confidence bounds)

Patient characteristics are presented in Table 1. A total of 3870 comparative data points were obtained from the a-line and CT device for the 30 min time window comparison. For the data set collected during the entire procedure, 58701 comparative data points were obtained, spanning approximately 114.5 h. Across the 24 subjects, the percentage mean of excluded data was 2.8% (SD: 4.0, range: 0–12.7%) while the median was 1.0%. The 30-min study period results are presented as correlations and Bland-Altman graphs for MAP, systole and diastole in Figs. 3, 4 and 5. The correlation between the a-line and the CT device for MAP, systolic and diastolic were 0.92, 0.86, 0.91, respectively (*p <* 0.0001 for all). The Bland-Altman comparison yielded a bias (as measured by overall mean difference) of −0.57, −2.52, 1.01 mmHg for systolic, diastolic, and mean arterial pressures, respectively with a standard deviation of 7.34, 6.47, 5.33 mmHg for systolic, diastolic, and mean arterial pressures, respectively (*p <* 0.001 for all comparisons). The corresponding results for data collected during the entire procedure (58,701 data points) including the 30-min study for MAP, systolic and diastolic were 0.87, 0.89, 0.82, respectively (*p <* 0.0001 for all the comparisons). Bland-Altman comparison for MAP, systole and diastole over the entire length of the procedures were SD 9.73, 13.13 and 10.23 mmHg, respectively (*p <* 0.0001 for all the comparisons).

**Table 1:** Patient Characteristics

	*N = 24*
Age (years)	
Mean (SD)	67 (10)
Range	46–83
Gender, n (%)	
Male	13 (54)
Height (cm)	
Mean (SD)	166.6 (12.9)
Range	140–185
Weight (kg)	
Mean (SD)	73.1 (17.0)
Range	45–99
BMI	
Mean (SD)	26.6 (6.9)
ASA status	
II/III/IV	3/19/2
Procedure	
Pancreaticoduodenectomy	19
Other	5
Comorbidities (%)	
Hypertension	14 (58)
Coronary artery disease	3 (13)
Peripheral vascular disease	4 (17)
Chronic obstructive pulmonary disease	9 (38)
Diabetes	9 (38)
Renal disease	4 (13)
Patients requiring vasopressor support	24 (100)

SD = standard deviation

**Fig. 6:**
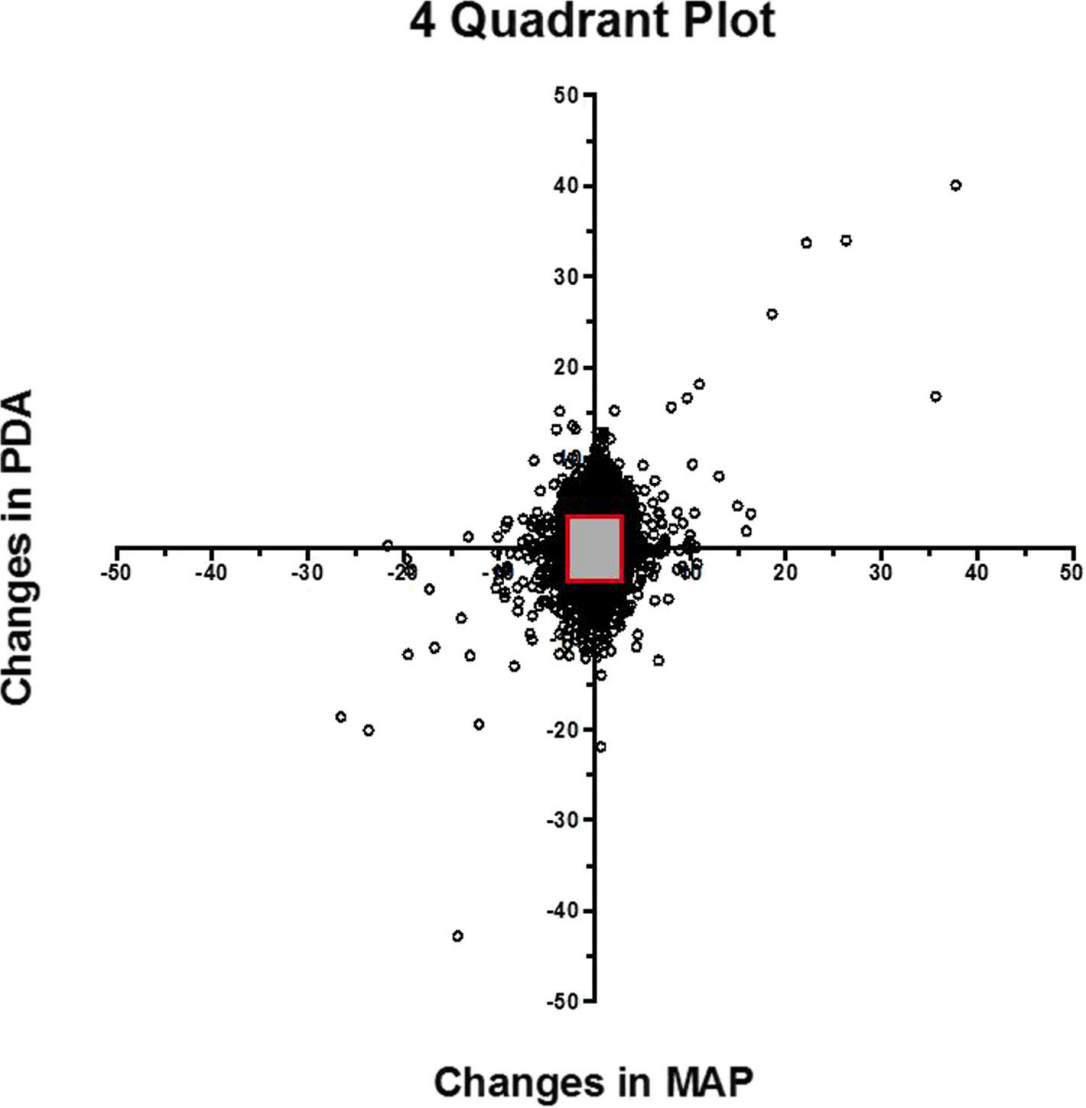
4Q plot of the consecutive changes in the A-line vs. the consecutive changes in CareTaker The 10% zone of inclusion is included

**Fig. 7:**
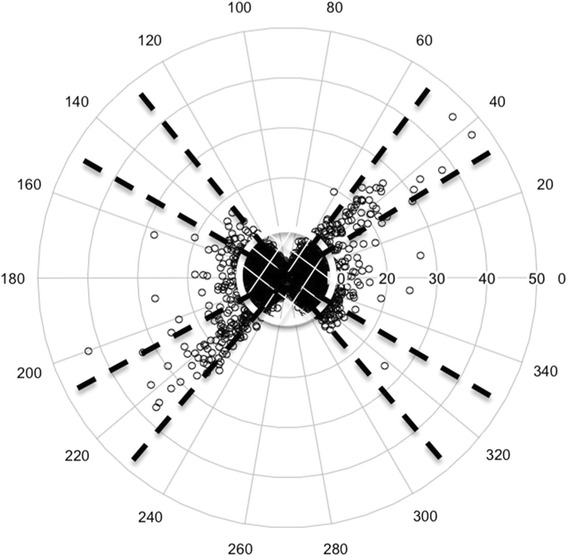
Polar plot examining the trend and confidence bounds of the difference between the A-line and the CareTaker

**Fig. 8:**
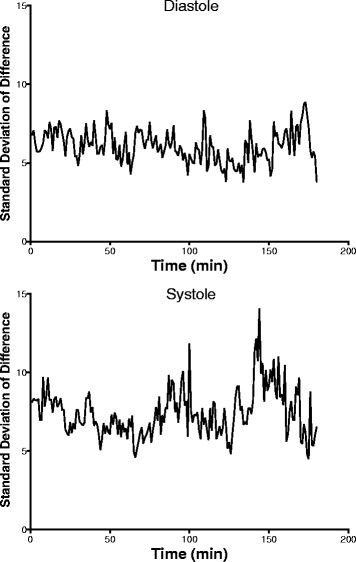
Standard Deviation of the differences between a-line and CareTaker data for all patients

To measure the trending agreement and bias between the CT and a-line data, 4-Quadrant and polar plots were calculated. The 4-Quadrant plot (Fig. 6) displays the successive differences during the 30-min study period. There is 99% data concordance comparing consecutive differences less than 10 and 95% concordance comparing consecutive differences less than 5, for which both devices measured the same direction of change in measurements. A polar plot examining the trend between the a-line and the CT show most points falling within the confidence bounds at 31.5°/52° of the plot, corresponding to, the 90%/95% confidence intervals, respectively (Fig. 7). Over 99% of the points on the polar plot are within the 95% confidence bounds. Additionally, there is good agreement between the devices and no evidence of any drift over the full time period. The standard deviations of the differences at all time points and patients are within 4–8 mmHg and 4–14 mmHg for diastole and systole, respectively (Fig. 8).

In order to quantitatively assess the dynamic range of the comparison blood pressure data, the intra-patient maximum minus the minimum systolic and diastolic pressures from the a-line were compared for each 30 min comparison window. The mean ranges for systolic, diastolic and mean arterial blood pressures from all patients were 35.1 mmHg (SD = 20.6 mmHg), 18.3 mmHg (SD = 9.0 mmHg) and 23.9 mmHg (SD = 12.3 mmHg) respectively. No significant between patient variability was observed for any of the measurements using general linear models of the difference between measurements and average measurement controlling for time of the measurement during surgery.

## Discussion

There is a significant need for continuous, accurate, and precise non-invasive blood pressure (cNIBP) monitoring in acute care patients. Lack of precision and accuracy has been the primary impediments to a wider acceptance of several cNIBP measurement methods. The new CT device investigated here uses pulse contour analysis of the arterial pressure pulse acquired with a hydrostatically coupled sensor system as a means to track blood pressure beat-by-beat. We show here that the CT device is a precise and accurate instrument and can be an attractive alternative for non-invasive measurements.

This study was conducted to determine what, if any, difference may be between the CT and a-line values. Therefore, because the estimation of the difference between the methods was the outcome of interest, no power analyses for sample size estimates were calculated prior to the study. The final cohort size of 24 was determined primarily by patient availability and is 1.6 times larger than the required size of 15 patients using the AAMI standard when an a-line is used for comparison (http://my.aami.org/aamiresources/previewfiles/8106002_1306_preview.pdf). A post-hoc power analysis was calculated using a repeated measures analysis to validates that 24 patients corresponds to power greater than 80% to detect differences of 10% at the 0.05 level.

The new CT and traditional a-line devices had comparable MAP, systole and diastole values for the measured procedure during the matched 30-min interval. Specific predefined performance requirements regarding accuracy and precision of non-invasive continuous devices are not yet explicitly defined. In this study, we use the AAMI standards; however, this standard was not developed for this specific purpose. While the standard is not intended for continuous blood pressure monitors and excludes dynamic blood pressure episodes as a basis for validation, it is the only applicable standard and for this dataset. Currently all FDA-approved blood pressure monitors utilize this standard as a basis for their approval [13–16]. We have included the Bland-Altman, 4-Q, [10] and Polar plot [11, 12] for our analysis and compared our results to the AAMI standards. The results for the 30-min comparison period fall well within the requirement of the AAMI standard that states that bias should not be greater than 5 mmHg and standard deviation should not be greater than 8 mmHg when using the Bland-Altman analysis. The further analysis using 4-Quadrant and polar plots confirm these results and show little to no bias and good concordance.

The comparison method applied here applies more stringent criteria than the AAMI 81060 standard prescribes. The standard calls for a reference reading to be obtained by collecting data from the a-line for at least 30 s before the reading from the device under test, and for at least 30 s after the reading from the device under test. If this range of the reference exceeds 20 mmHg for systole or 12 mmHg for diastole, the reference range is excluded from consideration [17]. Otherwise the range of the reference is defined as ±1 standard deviation around the mean value of a-line values, for both systolic and diastolic blood pressure values. If the reading of the device under test falls within the defined range of the reference, the difference between device under test and reference is defined as 0 mmHg, which is the reason the range is referred to as the zero-zone [18]. For values outside the range, differences are calculated by taking the difference between the reading and the applicable edge of the range. The mean of the differences cannot exceed 5 mmHg and the standard deviation cannot exceed 8 mmHg. Since the comparison here was between two values, as opposed to a value and a range, the methodology applied exceeded the requirements of the ANSI/AAMI/ISO 81060–2:2013 [17].

With two devices used over the time period, there is a risk for baseline drift and deviations in the measurements. To evaluate the change in performance over time, we plotted the standard deviations of the difference between CareTaker and the a-line values over time (Fig. 8). However, comparing the standard deviation over time between the two devices does not reveal any significant drift or pattern over the whole measurement period. These small changes will be further reduced in clinical settings where re-calibrations will reasonably occur in 30 min intervals, a point that has been made in the context of other studies [8]. The issue of re-calibration is also addressed by the fact that the next version of the CareTaker will be capable of self-calibration, either automatically in response to significant sensed hemodynamic changes or on demand.

With regard to the differences evident between the a-line and CT, a contributing factor may have been resonance artifacts, that compromised the fidelity of the intra-arterial waveforms. These artifacts, specifically under-damping, may lead to clinically relevant differences between actual and displayed pressure values. The impact of underdamping typically has the greatest effect on systolic pressure and the least on diastolic [19–21]. Further issues derive from its physical attributes as it can be knocked or fall off a patient, which may impact the readings. There is also some minor training for initial use of the device and its computer. These issues can be resolved with proper training and increased familiarity and should not impact its measurements.

Some potential limitations of this study include the lack of severely hypotensive patients and the inability to perform sub-group analysis based on various clinical patient parameters and demographics. Future studies should examine the effects of age and various clinical conditions such as heart failure, peripheral vascular disease, arteriosclerosis, diabetes, significant blood loss and hyper/hypotension on the non-invasive measurements obtained with the CT device. The effect of low perfusion in the digits due to cold or other reasons should be also be investigated. Limitations of the current device include the inability to use it in the young pediatric population due to size of the finger cuff and the need for calibration of the blood pressure. Recalibration is also required with significant changes hemodynamics and arterial wall stiffness.

The CT device has the potential to replace a-line measurements for accuracy and precision, but as it is a new modality, it has novel issues and will require further validation before larger scale use. One of the CT device’s central practical benefits is the comfortable data acquisition using the finger cuff, increasing potential ease of use and patient comfort. The unique feature for the device is the PDA model that is based on a concrete physical model that explains the structure of the peripheral arterial pressure pulse due to central arterial reflection sites [3, 6]. In addition to monitoring blood pressure, modeling the superposition of the component pulses makes it possible to explain and predict otherwise confounding pulse envelope changes. As such, the continuing development and refinement of the PDA method may also contribute to the understanding of the structure of the peripheral arterial pressure pulse. Information that can potentially be derived besides blood pressure are age and disease related changes to arterial stiffness.

## Conclusion

We have presented evidence that the non-invasive tracking of arterial pressure using the Pulse Decomposition Analysis pulse analysis approach is possible within the guidelines of the ANSI/AAMI 81060 standard. Comparison values were obtained over considerable blood pressure ranges as a result of hemodynamic challenges due to abdominal surgery, supporting the feasibility of this non-invasive and non-intrusive approach to hemodynamic monitoring.

## Abbreviations

AAMI: Association for the advancement of medical instrumentation
a-line: Arterial line
cNIBP: Continuous non-invasive blood pressure monitors
CT: CareTaker®
MAP: Mean arterial pressure
PDA: Pulse decomposition analysis
SD: Standard deviation
SNF: Signal/noise factor
